# Psychotropic drugs in Nepal: perceptions on use and supply chain management

**DOI:** 10.1186/s12992-018-0322-4

**Published:** 2018-01-24

**Authors:** Nawaraj Upadhaya, Mark J. D. Jordans, Dristy Gurung, Ruja Pokhrel, Ramesh P. Adhikari, Ivan H. Komproe

**Affiliations:** 1 Transcultural Psychosocial Organization Nepal, Kathmandu, Nepal; 2 Department of Research and Development, HealthNet TPO, Amsterdam, the Netherlands; 3 Centre for Global Mental Health, Institute of Psychiatry, Psychology and Neuroscience, King’s College London, London, UK; 4 Faculty of Social and Behavioural Sciences, Utrecht University, Utrecht, the Netherlands

**Keywords:** Psychotropic drugs, Nepal, Supply chain management, Mental health

## Abstract

**Background:**

Psychotropic drugs play an important role in the treatment of mental, neurological and substance use disorders. Despite the advancement of the use of psycho-pharmaceuticals in the developed countries, the psychotropic drug production and supply chain management in low- and middle- income countries are still poorly developed. This study aims to explore the perceptions of stakeholders involved in all stages of the psychotropic drug supply chain about the need, quality, availability and effectiveness of psychotropic drugs, as well as barriers to their supply chain management.

The study was conducted among 65 respondents from the Kathmandu, Chitwan and Pyuthan districts, grouped into four categories: producers, promoters and distributors (*N* = 22), policy makers and government actors (*N* = 8), service providers (*N* = 21) and service users/family members (*N* = 14).

**Results:**

The respondents reported that psychotropic drugs, despite having side effects, are 1) needed, 2) available in major regional centers and 3) are effective for treating mental health problems. The stigma associated with mental illness, however, forces patients and family members to hide their use of psychotropic drugs. The study found that the process of psychotropic drug supply chain management is similar to other general drugs, with the exceptions of strict pre-approval process, quantity restriction (for production and import), and mandatory record keeping. Despite these regulatory provisions, respondents believed that the misuse of psychotropic drugs is widespread and companies are providing incentives to prescribers and retailers to retain their brand in the market.

**Conclusions:**

The production and supply chain management of psychotropic drugs is influenced by the vested interests of pharmaceutical companies, prescribers and pharmacists. In the context of the government of Nepal’s policy of integrating mental health into primary health care and increased consumption of psychotropic drugs in Nepal, there is a need for massive education and awareness as well as strict monitoring and supervision to avoid the misuse of psychotropic drugs.

**Electronic supplementary material:**

The online version of this article (10.1186/s12992-018-0322-4) contains supplementary material, which is available to authorized users.

## Background

Psychotropic drugs play an important role in the treatment of mental, neurological and substance use (MNS) disorders [1] and therefore availability of, and access to, these drugs have been considered vital in mental health care [2]. Psycho-pharmaceutical advancements could increase the effectiveness and efficiency of mental health treatments, but this potential has not been realized in low and middle income countries where treatment for MNS disorders is inadequate [3]. Many people with MNS disorders in low and middle income countries remain untreated because of inadequate access to psychotropic medicines [4], especially for those disorders such as psychosis and bipolar disorder for which psychotropic drugs are the first line of the treatment. To reduce this treatment gap, the World Health Organization (WHO) has recommended the implementation of the Mental Health Gap Action Program (mhGAP) in primary health care settings [5]. However, interventions with psychotropic drugs are different from psychotherapy or case management, and involve greater risks of inappropriate use and unintended consequences (side effects and further complications). Therefore, in the context of psychotropic drugs being included in the mhGAP intervention in primary care settings, it is important to assess the entire psychotropic drug cycle from production to consumption. This is because the access, utilization and efficacy of psychotropic drugs are affected by both the perception of the people using the drug as well as the organizations and actors involved in drug production and supply chain management. Although drug efficacy is evaluated by pharmacological action, studies have shown that this one-dimensional perspective is insufficient; the ‘real impact of the medicines’ is connected to several interwoven dimensions of individual biology and socio-cultural dynamics [6]. Therefore, psychotropic drugs can have several meanings for people who use them [7], and can have different effects (contrary to the biomedical principle that psychotropic drugs prescribed in certain doses have identical effects in all patients) depending on the illness experiences of individuals and how they interpret the effectiveness of psychotropic drugs in the context of their lives [6]. In high income countries, adequate access to psychotropic drugs is achieved by sustainable financing and reliable supply systems [4], whereas low- and middle- income countries (LMICs) have inadequate finance and supply systems thus limiting the access to psychotropic drugs. Additionally, in low- income countries little awareness of mental health problems and socio-culturally constructed explanatory models of illness and treatment pathways determine which service to access. Similarly, the prescribing behaviour and consumption of drugs as per the prescription are affected by the socio-cultural context and belief systems of stakeholders. As a consequence, the attitude of the society towards mental illness influences the help-seeking behavior. In Nepal, due to stigma associated with mental illness, many people hesitate to seek services and do not arrive to health facilities for the treatment of their mental health problems. This affects the demand and supply of psychotropic drugs. The production of drugs follow protocols that primarily focus on cost effectiveness and drug efficacy, whereas drug distribution, prescription and use is less structured and more influenced by the perspectives of people involved [8]. This is important to consider because the meaning and expectation the client associates with the use of psychotropic drugs has a significant impact on the effectiveness of the drug.

In Nepal, psychotropic drugs are available from Nepalese and Indian pharmaceutical companies and are regulated under the Narcotic Drugs (Control) Act 1976. Within the Ministry of Health (MoH), the Department of Drug Administration (DDA) established in 1979 is responsible for regulating import and production, as well as monitoring, distribution, prescription and record keeping of psychotropic drugs in Nepal. Guided by the drug policy of 1995, the national list of essential medicines, Nepal (2011) includes 15 psychotropic drugs and the free drug list approved by MoH in 2014 includes five psychotropic drugs (as given in Additional file 1: Table S1). Despite these policy commitments to make psychotropic drugs available in Nepal, access to psychotropic medicines in much of the country is still limited. This problem is compounded by a dearth of literature on supply chain management of psychotropic drugs, therefore the barriers to improving mental health care access are not well understood. To improve psychotropic supply chains, the barriers and the enablers in current supply chains need to be identified and properly addressed. One study on pharmaceuticals in South Asia included an anti-depressant drug, fluoxetine [9] used in Nepal. However, this research did not include any other psychotropic drugs. Furthermore, no studies from Nepal have described the perceptions of people involved in the entire psychotropic drug supply chain.

To address this knowledge gap and provide input for the adequate roll out of the mhGAP intervention in Nepal’s primary health care, our study aimed to explore the perceptions of stakeholders (involved in all stages of the psychotropic drug supply chain from production to consumption) about the need, quality, availability and effectiveness of psychotropic drugs in Nepal as well as the barriers to effective psychotropic drug supply chain management.

## Methods

### Setting

This study was conducted in the Kathmandu, Chitwan, and Pyuthan districts of Nepal. Kathmandu, the capital city, was selected specifically in order to include policy makers and government agencies involved in the supply chain management of drugs. Chitwan and Pyuthan both were purposively selected because a government-Non Governmental Organization (NGO) model mental health program was being implemented in these districts. The program for improving mental health care (PRIME) in Chitwan and the mental health beyond facilities (MHBF) project in Pyuthan involved training primary health care staff on management of psychotropic drugs and basic counseling as part of a comprehensive District Mental Health Care Plan, based on WHO’s mhGAP [10]. The services ranged from community mobilization, family support, psychosocial counseling and psychotropic medication. Chitwan represented a district with more advanced medical facilities, having a psychiatric ward in the government regional hospital and private medical colleges, whereas Pyuthan represented a hilly rural area with limited health facilities.

### Sampling

For the study, the sample (*N* = 65) was conveniently selected through a process of purposive and snowball sampling, identifying people involved at any stage of the psychotropic drug supply chain in Nepal. The sample was divided into four major categories, (a) producers, promoters and distributors; (b) policy makers and government actors; (c) service providers; and (d) service users and family members. The respondents for category “a” (*N* = 22; 9 from Kathmandu, 9 from Chitwan and 4 from Pyuthan) included people involved in the production, import, dispensing, and promotion of psychotropic drugs. For category “b” the respondents (*N* = 8; 6 from Kathmandu and 2 from Chitwan) were government planners and policy makers working in ministry of health, its departments in Kathmandu, and district public health office, Chitwan. The respondents for category “c” (*N* = 21; 3 from Kathmandu, 9 from Chitwan and 9 from Pyuthan) included health workers (both specialists such as psychiatrists, psychiatric nurses and generalists such as medical officers and primary health care workers) providing mental health services in the study locations. The category “d” respondents (*N* = 14; 8 from Kathmandu, 5 from Chitwan and 1 from Pyuthan) included people who had used psychotropic drugs for the past six months and their family members.

### Data collection instruments and process

The template for semi-structured interview “topic guides” was constructed for the above-mentioned categories of respondents. The major areas of focus for the questions were: processes involved in supply chain management, perceptions of need and use of psychotropic drugs, challenges in supply chain management, relationships with other stakeholders in the supply chain management, and recommendations for effective management of psychotropic drugs. The “topic guides” were pilot tested through interviews with a member from each category in a non-study district in Nepal. Based on the results of the pilot interviews, the questions were simplified, their sequence were re-ordered and some probing questions were added.

The data collection took place between September 2013 and March 2014. Data collection for service users was conducted during home visits and health center visits, while interviews with respondents from other categories were conducted through work place visits. The respondents were asked to choose the place for the interview to ensure privacy and confidentiality, as well as to make sure that they would be at ease and could respond freely, comfortably and honestly. Where possible, the researchers also observed the interaction between patients and retailers, patients and prescribers, marketing representatives and retailers. These observations were noted and written-up as field notes on the same day. All interviews were conducted in the Nepali language by a team of six experienced Nepalese researchers. The researchers had participated in a one-week workshop which helped them to familiarize themselves with the research design and methods, and to update their understanding on qualitative data collection processes. During the workshop, mock interviews and role plays were conducted for researchers to assess their comprehension of the topic guides. Data was collected through digital audio recordings, note taking, and field observations. The respondents were informed about the nature, objectives, and use of the study, and verbal consent was obtained from each respondent after the assurance of confidentiality.

### Data analysis

Thematic analysis was used in the data analysis process. Researchers transcribed the interviews immediately after meeting the respondents. The transcripts were translated into English by professional translators and checked by the research supervisor. The translation was necessary as the data needed to be shared with the research team members of an international consortium project called Emerging Mental Health Systems in Low- and Middle- Income Countries (Emerald), of which this study was a part. The Emerald project aims to strengthen the systems and processes that are necessary for effective mental health service delivery [11]. Ten percent of the interviews, along with field notes and observations, were randomly selected and read separately by four researchers, who identified, compared, and discussed broad themes from the data. On the basis of previously discussed themes, two researchers (DG and RP) separately coded 35% of the data manually, and the new themes that emerged during the coding process were added. The codes were discussed with research team members to evaluate comparability and reliability between researcher identified themes, and a coding framework with themes and sub-themes was developed. The finalized coding framework was subsequently applied to the entire dataset using the qualitative data analysis software, NVivo 10.

## Results

The findings are grouped under the broader themes ‘processes’, ‘perceptions’ and ‘barriers/issues’. In this study, respondents’ comments referred to all mental disorders and psychotropic drugs available and used in Nepal.

### Psychotropic drug supply chain in Nepal

**Fig. 1:**
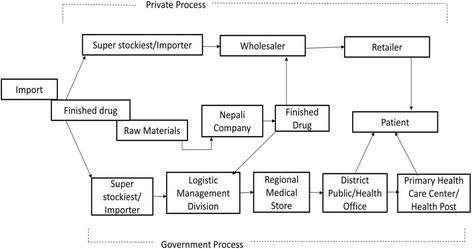
Process of psychotropic drug supply chain management in Nepal

As depicted in Fig. 1, according to the respondents, the process of psychotropic drug production starts with the import of raw materials; this is because these materials are not available in Nepal. With prior approval from the Department of Drug Administration of the Ministry of Health and Counter Narcotic division in the Ministry of Home Affairs, Nepalese pharmaceutical companies import raw materials mainly from India. As per the specimen approved by the DDA, Nepalese companies produce psychotropic drugs and send to the wholesalers (also known as stockest or distributors). For drugs produced outside of Nepal, the company’s agent in Nepal (also called a ‘super-stockiest’) needs to register the imported drugs with the DDA and obtain the approval for the quantity of drugs to be imported each time. All pre-approval documents from the DDA should be submitted to the Ministry of Home Affairs for final approval. In the private sector, retailers place an order with the wholesalers and the wholesalers dispatch the medicine to retailers via transport companies. In the government sector, companies (or their agents, in the case of foreign companies) are selected to provide medicines through a tendering process. The medicines are procured either centrally or at the district level. The district level procurement is small in quantity and is meant to meet the demand of drugs in accordance with patient flow. For the central level procurement, the Primary Health Care Revitalization Department (PHCRD) is responsible for the planning and budgeting of drugs under the free drug list. Some psychotropic drugs are included in the free drug list so PHCRD provides the list of drugs to be procured to Logistics Management Division (LMD), which then procures and distributes through five Regional Medical Stores (RMSs). The RMSs supply the medicines to District Hospitals and the District Public Health Office based on demand. When the medicines are procured centrally and distributed through RMSs, it is called the “push” system and when the medicines are procured at the district level to meet the increased demand, it is called “pull” system. According to the respondents, the supply chain management process of psychotropic drugs is similar to all other general (non-mental health) drugs, but extra precautions and restrictions are imposed because of the chances of being misused, during import, production, transport, storage and distribution. For example, to limit the chances of misuse, all psychotropic drugs are separated from other general drugs and stored in a locked cupboard to which only authorized persons have access. According to the rules set by the DDA, the psychotropic drugs cannot be sold or bought without prescription. The expired drugs are returned to the company through wholesalers, or disposed of by burning and burying the medicines.

### Perceptions on need, quality, availability and use of psychotropic drugs

Table 1 provides a summary of respondents’ perceptions per category, while the narrative that follows focuses on overarching perceptions held amongst all respondent categories.

**Table 1:** Perceptions on need, quality, availability and use of psychotropic drugs

Stakeholders	Need	Quality / Effectiveness	Availability	Use/stigma
Producers, promoters and distributors	Medicine along with other alternative healing.	Both drugs produced in Nepal and imported from outside are of good quality and are effective.	Available where mental health services are being provided.	There is negative societal attitude towards people who use psychotropic drugs.
Policy makers and government actors	Yes for severe cases only, for other cases as a last resort.	Drugs are of good quality, although people believe that foreign drugs are more effective.	Available at the district level where mental health services are available.	There is stigma for people with mental illness which affects the access and use of psychotropic drugs.
Service providers	Medicine for severe cases, counseling and other therapy for minor cases.	Some medicines produced in Nepal are of low quality and less effective.	The availability of drugs have increased drastically, but not to all areas of Nepal.	There is not only stigma for those who use psychotropic drugs but also to those who provide mental health services.
Service users and family members	For severe cases only Medicines with other therapies are needed.	Medicines are of good quality and are effective.	Drugs are available at the district or at regional centers only.	There is huge stigma for those who use psychotropic drugs. Consequently, patients and family members try to hide as much as possible.

#### Need of psychotropic medicines for the treatment of mental health problems

Most of the respondents were of the opinion that psychotropic drugs are needed to treat people with mental illness but that drugs alone were not the preferred treatment. Most respondents emphasized the importance of counseling, a supportive family environment, and other behavioral and psychotherapies along with the medications. A medical doctor from Chitwan said, *"both counseling and medicine usage are equally important for patients with mental health problems. Patients need counseling about the medicines too"*. A service user from Kathmandu agreed with the notion of an integrated approach by saying: "*other secondary treatment methods like cognitive behaviour therapy and other behaviour therapies should be done. Only taking medicines is not good*".

The psychiatric nurses were of the opinion that drugs were necessary only for individuals with severe mental health problems. A nurse from Chitwan elaborated this by saying, "*if the problem is psychosomatic then drugs are not required. Counseling and an understanding family are better. But in chronic cases, drugs are necessary. For instance, [cases of] Schizophrenia, MDP (Manic Depressive Psychosis), Severe Depression need medication"*.

Some respondents said that drugs should be used as a last resort when other methods have not worked. This is evident by the following excerpt from a government officer:

"*If there is no such environment, or if there is no family or social support, or if the social or family support doesn’t help the problem, then as a last resort there is always medication"*.

The medical representative and psychiatrist from Kathmandu, however, placed emphasis on medication as the primary focus, supplemented by other interventions, such as family support:

"*medicine is the most important, followed by family support and understanding*".

The service users also agreed on the need for drugs but said that doctors prescribe drugs even when there is no need. A service user from Kathmandu said, "*we should definitely give medicines to severe psychotic patients. They [doctors] prescribe medicines for such mild depression also, so that the patients get lifelong dependency [on drugs]. Due to this, the company also has lifelong business and doctors also get more money, this is the situation here*".

#### Quality and effectiveness of psychotropic drugs

Nepalese producers reported that their product was of good quality as they are following the WHO good manufacturing practices guidelines and quality checks of raw materials and the finished product are regularly done. A producer from Chitwan said, "*we have a quality control laboratory too. This lab tests the quality of the raw materials which have been imported or bought.....when the right quantity comes, we check for the right quality as per specification*". Despite this, the prescribers and the general public thought that the foreign drugs are better quality and more effective than those produced in Nepal. A Kathmandu-based psychiatrist expressed a similar sentiment, he said, "*most of the drugs from Nepali companies don’t work, but the same drug from an Indian company with the same dose, does work*".

A producer from Kathmandu said that the drugs produced in Nepal are of a high quality and can compete with the drugs imported from outside of the country. He said, *" there is not such difference between the drugs produced here and those imported from abroad, they are similar. We are also able to give better quality"*. A Kathmandu-based medical representative also said "*initially medicines from Indian companies were widely used, but these days prescription of Nepalese drugs has increased*".

The service users and caregivers felt that the psychotropic drugs were effective. A service user from Chitwan said, "*I have a positive view regarding it[drug]. The medicine is doing me good*". A similar perception was held by another service user who said, " *my view about the medicine,[is that] it is good. It has cured me. What this medicine is doing to others is a different matter. I say that this is doing me good*".

A caregiver from Kathmandu had positive remarks on the effectiveness of psychotropic drugs and said, *" the medicine is really effective. If there hadn’t been any doctors or medicines…then I would have faced a lot of challenges. I strongly believe that the medicine works and that it should work for everyone"*.

A psychiatric nurse from Chitwan was of the opinion that drugs were effective when taken properly and when other supportive aspects were in place. She said, "*psychotropic drugs work well for patients if they take them regularly... If we can combine diet, counseling and drugs then it helps. There is no benefit if we take psychotropic drugs as a typhoid drug*".

Though most of the respondents said that psychotropic drugs were effective, the side effects of the drugs were mentioned mainly by service users, care givers, doctors and nurses. According to a Chitwan-based psychiatrist, "*any [psychotropic] medicine might cause a hypersensitivity reaction...for example rashes, flushing, sweating, increased rate of palpitations etc*". Weight gain was another common side effect of psychotropic drugs, as explained by a service user from Kathmandu: "*this medicine has increased my weight, actually I was not this fat. The medicine is making me dull. This is hindering my creativity, and harming my personality*".

Some prescribers mentioned that the government’s free drugs list contains old psychotropic drugs that have more side effects than the new ones available on the market. The side effects of psychotropic drugs were cited by some respondents as one of the reasons for mental health patients discontinuing the medication, and accounts for a higher rate of relapses.

#### Availability of psychotropic drugs

Most of the respondents were of the opinion that, compared to the past decade, the availability of psychotropic drugs had drastically increased. A psychiatrist from Chitwan said, "*ten years back when I came to Chitwan there were no drugs and it was very difficult....we used to call the company and ask them to bring the drugs....before there was no availability of drugs but slowly now all the medicines are available*". Although psychotropic drugs are not available in large parts of Nepal, the Additional file 1: Table S1 shows that there are quite a number of psychotropic drugs available in the Nepalese market, mainly in the capital and regional centers.

#### Use of psychotropic drugs

The use of psychotropic drugs, especially in rural parts of Nepal, is affected by multiple factors. Some of these factors include low help seeking behavior due to lack of awareness about treatment facilities for mental health, the lack of health workers trained and authorized to prescribe psychotropic drugs, lack of mental health services, unavailability of psychotropic drugs and stigma associated with mental illness. Mental health problems and the use of psychotropic medications are both highly stigmatized in Nepalese society. Patients often hide and do not disclose that they are taking psychotropic medication. A psychiatrist from Kathmandu shared his experiences, " *patients who have anxiety or depression … they want to discontinue the medicine as soon as possible. It is because they don’t want others to know about their problems and that they are taking psychotropic medicine*". Family prestige and cultural problems in getting children married were some of the reasons for not disclosing mental health problems. "*He* [father] *then said that if anyone in the village had come to know that she was taking medicines for such an illness, none of the men would have agreed to marry her* ".

According to a service user from Chitwan, people in the community dominate and behave badly towards those taking psychotropic medicines, "*their view is, like I can’t earn my living. They say that I can’t work and earn, I should beg with a bowl. Some of them also say that I am a burden to this earth*".

Due to this high level of stigma, socialization for people taking psychotropic medication is a challenge, as expressed by a service user from Kathmandu, "*right now, I feel good being here [treatment center]. I don’t think I will be fully able to socialize outside though. However, I am trying to socialize*". Sometimes, even when community members are supportive, patients using psychotropic drugs and their family members feel inferior and experience perceived stigma. A caregiver explained: "*I don't have any difficulties but I feel in my heart. Others have not said anything but it breaks my heart that my son has got this problem..*.".

### Perceptions on barriers to the effective psychotropic drugs supply chain

Table 2 provides the perception of stakeholders on major issues and barriers to effective psychotropic drug supply management in Nepal which is substantiated in the following sub-themes and narratives.

**Table 2:** Perceptions on barriers to effective supply chain management of psychotropic drugs

Stakeholders	Barriers to effective supply chain management of psychotropic drugs
Producers, promoters and distributors	• There is market competition. Need to spend money for bonuses and incentives. There is not much profit in psychotropic drugs but there are more hassles in its production, import and record keeping. • The drugs imported by one agent cannot be imported by another agent despite high market demand for that drug. • There is substitution of drugs by retailers due to higher bonuses and incentives by other companies. • Nepal is dependent on India for raw materials which is a challenge for independent manufacturing of psychotropic drugs in Nepal.
Policy makers and government actors	• The drugs are prescribed in brand names with the hidden motives for incentives. • The storekeepers at district level have limited knowledge on drugs as they do not have medical background. • The inappropriate use or misuse of psychotropic drugs could increase if prescription authority is provided to primary health care workers without proper training and supervision. • The supervision and monitoring of psychotropic drugs at the district level is minimal so there are chances of misuse.
Service providers	• Old generation psychotropic drugs listed in government’s free drug list have lots of side effects compared to the new generation drugs available on the market. • Some patients overdose on the drugs while others refuse to take psychotropic drugs due to side effects. • There are limited health workers in the district who can effectively diagnose and prescribe psychotropic drugs. Many medical officers do not feel confident in prescribing psychotropic drugs. • Patients are sometimes used by drug addicts to get the psychotropic drugs.
Service users and family members	• Psychotropic drugs are effective but they have lots of side effects. • Due to stigma it is difficult for patients and family members to share that they are using psychotropic drugs. • Psychotropic drugs are not available at the community level, therefore, patients have to travel far distances to buy the drugs. • Doctors focus more on medication even for cases that could be managed by counselling and other social support interventions.

#### Prescribing authority

Among the respondents there were divergent views regarding whether the authority to prescribe psychotropic medication should be given to primary health care workers such as Health Assistants (HAs). Those who spoke in favour of it said that, in many remote areas, there are no doctors and that it is the HAs who provide health services, therefore it would be better to give the prescribing authority to HAs. A district level policy maker from Chitwan said, "*psychiatrists and MBBS doctors are not available in all areas hence up to PHC [Primary Health Care] and Health Post Level, if the HA is provided with the rights[to prescribe] then it would be better*". Those who were in favour for HAs to have the responsibility of prescribing psychotropic medicines stressed the importance of proper training and supervision to avoid misuse and mismanagement.

However, respondents who opposed HAs having these responsibilities thought that if paramedics who have not studied pathology in detail were given prescribing authorities there would be more chance of misuse or inappropriate use. A MBBS doctor said, "... *the ones who study HA they have not studied pathology. On that basis, they should not be allowed* [to prescribe]". A senior psychiatrist was of the opinion that even the MBBS doctors should not be allowed to prescribe, "*I think only psychiatrists should prescribe...even medical officers should not be allowed...people buy it as a general medicine...that is not good...we have to consider a lot before prescribing...*". On the contrary, a psychiatric nurse said "*even a HA can do it if he is given special training and if he is made understood all the things[related to psychotropic drugs]*...". Some thought that HAs should not have direct prescribing authority but could be given authority to provide repeat prescriptions. A policy maker from Kathmandu said, "*if the drugs have been prescribed by the specialists, then the HAs can follow-up on those cases and dispense the drugs based on those prescriptions*".

This shows that the prescribing authority is also a supply chain issue because when primary health care workers are authorized to prescribe psychotropic drugs there will be more demand for such drugs. More drug demand means more stakeholders will be involved in drug production and supply chain. Secondly, besides the service availability and demand for drugs, the prescription authority to primary health care workers will also bring the issue of brand named drug prescription and incentives/bonuses provided by the companies to the prescribers affecting the supply chain cycle for psychotropic drugs.

#### Practice of bonuses and commissions for psychotropic drugs

The respondents accepted that there is a widespread bonus practice in Nepalese pharmaceutical setting. The doctors are especially lured by companies to prescribe drugs from certain brand names. According to the respondents, the companies mobilize the Medical Representatives (MRs) to promote the product and to lure doctors and retailers. A psychiatric nurse said, " *we don't have meetings with them [MRs], they meet only the doctors, they give gifts to them and ask them to prescribe the medicine"*. A senior psychiatrist admitted that MRs try to influence doctors to prescribe certain drugs: " *before they [MRs] used to influence....but I don't think they have influenced me..... they give us pens,.... they give pamphlets, they bring calendars....what will they do[give] to us?....therefore I just see the effect of the drug*". One of the producers of psychotropic drugs also admitted to providing bonuses, but said that it is difficult to compete in the market because of high bonuses provided by the multinational companies. He said, "*the biggest challenge for now is 'bonus war'. We are not able to compete in bonus. There are high bonuses from Indian companies"*. The companies not only lure the prescribing doctors but also the retailers. In the words of a MR from Chitwan, "*instead of following the doctor's prescription, the companies go to the pharmacy and say, ‘ we will give one box free [when you buy] 10 boxes’, or ‘two boxes free [when you buy] 10 boxes’ and attract them and lure them*".

The practice of providing bonuses and offering commission is a barrier to the availability of effective drugs as the prescribers and retailers choose only those drugs that provide the most incentives, rather than the drugs that are higher in quality and effectiveness. Some producers reported that if they did not have to invest in chemists and doctors in the name of incentives and bonuses they would have made a lot of profit. According to them, currently the profit is not enjoyed by the production company but by middlemen (i.e. doctors and chemists/retailers).

#### Misuse of psychotropic drugs

Misuse of psychotropic drugs was acknowledged by most respondents. However, there were quite different views about who misuses these drugs and where misuse takes place. Some said that the misuse is by the paramedics and general practitioners because they prescribe medicines without having much knowledge about the drugs. A MR from Chitwan said, "*since the patient gets instant relief, the general practitioners are misusing these drugs [by overprescribing psychotropic drugs]*". In addition, drug addicts approach retailers ask for psychotropic drugs and misuse them. A pharmacist from Chitwan explained the behaviour of drug addicts when they come to the pharmacy, *" if they[drug addicts] come in a black jacket the first time then again after 2 hours they will come in a red jacket, the same guys*". Some retailers provided drugs to addicts out of fear or to make money. A MR explained this by saying, "*some friends are even providing such drugs under the table. As they get certain benefits from the sale of such medicines*". This argument was supported by a Kathmandu level policy maker who said, "*during inspections we found out that those drugs[psychotropic] were misused by some medical shops[pharmacies]. However, paracetamol is also misused by some people. So, it is not just the psychotropic drugs. In private pharmacies, if the drug users go to pharmacies and give them extra money, then they will get the drugs without prescriptions. It needs to be monitored more carefully*".

Most of the respondents thought that patients also misused the drugs by taking larger dosages than prescribed by the doctor. A psychiatric nurse said, ".... *they take an overdose of the same medicine and die...they think that it is better to die rather than take medicine daily*". According to the pharmacist from Kathmandu, the most commonly misused psychotropic drugs were Nitrazepam, Diazepam and Clonazepam.

#### Record keeping of psychotropic drugs

Most of the respondents from producer, promoter, wholesalers, retailer and prescriber categories said that they are aware about the record keeping provision for psychotropic drugs in a format prescribed by DDA. The format includes the name of the customer, the name of the prescribing doctor, the dose of medicine, the name of medicine, the date, and the quantity of medicine. There is also a provision that the customer should sign in on the register after they buy the medicines. A pharmacist from Kathmandu said, "*if we sell psychiatric and narcotic drugs, we get a prescription. We have to keep a record by writing the name of the patient and the name of the doctor who has prescribed the drugs*". However, most of the respondents (wholesalers and retailers’ category) admitted that record keeping was not being done properly. The retailers found it difficult to maintain the record keeping for psychotropic drugs; for them it took too much of their time in busy hours. Consequently some of the retailers stopped selling psychotropic drugs. A pharmacist from Chitwan said, "*as much as possible we try to follow that [DDA] format. But if there is a crowd you can see the problem [there is no time for record keeping], we try our best in those situations*". The monitoring mission of the DDA also found that only 50–60% of the pharmacies have completed the records.

## Discussion

The majority of the respondents thought that there was a need for psychotropic drugs especially for the treatment of severe mental health problems. However, many respondents also stressed that psychosocial counseling and other social support were equally important for the treatment of MNS disorders. Most of the respondents were of the opinion that the psychotropic drugs available in the Nepalese market are of good quality and effective for the treatment of MNS disorders. The service providers and service users agreed with this argument but also pointed out the many side effects that psychotropic drugs have, especially those enlisted on the government’s free drug list. Respondents who spoke about stigma said that there was a negative societal attitude towards people who use psychotropic drugs and therefore patients and family members try to hide from others that they are using drugs for their mental health problems.

There were diverse (contradicting) views about who misuses psychotropic drugs and where misuse takes place. Some respondents said that paramedics and medical officers with less knowledge of psychiatry misuse drugs by over-prescription. Some respondents were of the opinion that patients misuse by taking over-doses; while other respondents said that misuse happens mostly from the retailer’s side as they sell drugs unethically to earn a higher profit. The bonuses and commissions provided by the drug companies to prescribers and retailers were also thought to be responsible for the misuse of psychotropic drugs. In light of these findings, we later discuss issues related to the strict policy provisions but weak implementation, misuse of psychotropic drugs, bonus war, and provide some recommendations to address issues affecting the psychotropic drug supply chain management.

### Highly controlled on paper but freely available in practice

Many of the psychotropic drugs are subject to greater control/restrictions due to their potential for abuse [1]. Misuse can cause damage to physical as well as mental health, while inappropriate use can reduce the efficacy of the drugs resulting from non-compliance. In Nepal, compared to general drugs, psychotropic drugs have been highly controlled by law. For example, article 33 of drug act, 1978 has a mandatory provision of prescription and record keeping for psychotropic drugs [12]. Secondly, not only the Ministry of Health but also the Ministry of Home Affairs is involved in issues related to the psychotropic drugs. Despite these controls, psychotropic drugs continue to be misused [13]. The respondent from the DDA admitted that only about 50–60% of pharmacies have kept some form of record-keeping while, the rest have not done so at all. However, those who do keep records, many are not up to date. The lack of human resources at the DDA and the large number of medical stores to be inspected means that proper inspections are not possible so unethical practices and misuse are on the increase. Other possible reasons for misuse are the lack of implementation of existing regulations, poor quality assurance mechanisms and conflicts of interest between functions of the regulatory body which affect the availability of quality drugs [14]. Our study findings suggest an issue of trust among stakeholders due to the prescribers’ and retailers’ preferences for brand named drugs, frequent substitution of drugs in the market, and stakeholders’ diverse (contradictory) views regarding the quality of drugs produced in Nepal compared to those imported from India. The vested interest and lack of trust among stakeholders are barriers for the smooth and effective supply of psychotropic drugs in Nepal. Similar findings were reported by Brhlikova and colleagues while tracing three pharmaceutical drugs (Rifampicin, Fluoxetine and Oxytocin) in South Asia. They found that a lack of trust was a key issue that affected how drugs were produced, stored, distributed, prescribed and consumed. The authors also found that despite the Nepalese government releasing guidelines on the ethical promotion of medicines in 2007, the government was unable to instill confidence in the regulatory process. Consequently, beliefs about the effectiveness of the drugs were dependent on individual experiences and relationships rather than the government’s regulatory system itself [15].

In the context of increased competition between companies and numbers of pharmacies (retailers) selling psychotropic drugs, it has become a necessity to strictly implement psychotropic drug related policies and plans. The policy provisions regarding psychotropic drugs are promising, but their implementation is very slow. The non-compliance of legal provisions regarding psychotropic drugs is related to under-the-table economic benefits. Therefore, in order to reduce the misuse of psychotropic drugs, monitoring not only by the DDA but also from other professional entrepreneurial bodies, such as the Nepal Drug Dealers Association, the Nepal Chemists and Druggist Association, and the Civil Society Organization is urgently needed.

### “Bonus war”: A challenge for quality assurance and sustainability

The system of giving bonuses and gifts to prescribers in Nepal has promoted unethical promotion of drugs and tough competition amongst companies to replace each others’ products. The newer companies influence the doctors to prescribe their drugs over others [9]. Prescribing medication is much more than the mere act of writing the name of a drug on paper, it’s a social act, showing power and facilitates social control [8]. Therefore, prescribing is affected by the motives of several stakeholders involved in the production and supply chain. The higher percentage of drugs being prescribed under trade names also indicates how bonuses and commission play a role in psychotropic drug supply chain in Nepal. The drug utilization study conducted by Banerjee and colleagues in western Nepal showed that 88.1% of antipsychotic drugs were prescribed by trade names [16]. The retailers and prescribers we interviewed acknowledged that drug companies try to incentivize them in several ways by offering them gifts, free products and travel [9]. Due to these unethical practices, vested interests and influence of several profit motivated stakeholders, the ethical guidelines on drug promotion released by the Nepalese government in 2007 did not yield much positive results [15]. A review of the promotional brochures used by pharmaceutical companies in Nepal showed that brochures did not follow the WHO’s criteria for ethical medical drug promotion but emphasized a more commercial motive [17].

### Implications

First, like other drugs, the use of psychotropic drugs has been increasing in Nepal despite the negative attitude of the general community towards psychotropic drugs and psychiatric treatment, and the stigma associated with mental illness [18]. Due to fears of social discrimination many individuals and families hide that they are taking psychotropic medicines and sometimes they even discontinue the drugs to avoid stigmatization. Hence, anti-stigma programs at community, district and national levels are needed to give the message that mental health problems are like any other health problem and the use of psychotropic drugs is similar to the use of medicine for any other physical health problems.

Second, in the context of the government of Nepal’s policy of integrating mental health into primary health care [19] and psychotropic drugs being included in the Nepal government’s free drug list as well as in the WHO’s mhGAP intervention, there is a need for better understanding of how the supply chain of psychotropic drugs is managed, what the barriers are and how such barriers could be overcome. Our study findings suggest that the practice of bonuses and incentives in pharmaceutical products is common in Nepal which encourages unethical corrupt practices and misuse. This indicates that if bonuses and incentives could be regulated and misuse of the drug is controlled, then the current price of psychotropic drugs could be reduced. Cost-effective strategies such as an emphasis on prescribing generic names and quality monitoring from an independent authority [20] could be helpful in ensuring availability of high quality drugs at affordable prices which could ultimately facilitate the roll out of the mhGAP intervention in primary health care. Secondly, for the mhGAP to be adequately rolled out in community settings, there is a need to revise the current psychotropic drug prescribing authority, from medical officers to the primary health care workers. However, this change in prescribing authority should be accompanied with proper training, supervision and monitoring mechanisms to address possible inappropriate use or misuse of psychotropic drugs by primary health care workers.

Third, our study participants suggested that for the treatment of mental health problems not only drugs but also counseling and social support are necessary. This suggests that the stakeholders involved in the supply chain of psychotropic drugs, especially the prescribers, retailers and the patients need to consider carefully whether the drug they are prescribing, dispensing or using is absolutely necessary or whether a combination of drugs and counseling would be a better treatment approach. Previous studies have shown that when essential psychotropic drugs are provided along with psychosocial management strategies, such as individual and family counseling, the treatment becomes effective and thereby reduces disability and prevents relapse [4]. Drug therapy as well as psychosocial support are effective in treating mental health disorders such as depression, schizophrenia and alcohol dependence [3]. The importance of medication along with psychotherapy in the treatment of mental illness was evident from a hospital based study conducted in Western Nepal which showed that 51% of schizophrenia cases were given both drug therapy and psychotherapy [16]. As drug cycles start with production and end with consumption, it is important to consider the barriers related to the whole supply chain. The study findings suggest that stages such as prescription, dispensing and use of psychotropic drugs need greater attention when designing programs to address the challenges of psychotropic drug supply chain management in Nepal.

### Strengths and limitations

The mix of participants representing the government as well as the private sector involved in psychotropic drug production and supply chain management was a strength of the study. However, the number of participants in each group was fairly small, therefore, group comparisons of the views and perceptions of respondents was not possible. The sample included participants from the capital (Kathmandu), a regional center (Chitwan) and a rural district (Pyuthan) so the findings provide information on these three layers which would be important for future program planning and intervention design. However, the findings should be interpreted with caution as the study sample was not proportionate representation of members from Kathmandu, Chitwan and Pyuthan. In this study, we included all medicines used for mental health problems using the broadest definition of psychotropic drugs. Consequently, the findings from this study give an overview of psychotropic drugs in general and do not provide information on individual medicines used to treat specific disease conditions, such as anti-depressants or anti-psychotics. The decision to include all psychotropic drugs was due to the lack of research on the supply chain management of psychotropic drugs in general and our belief that a broader approach would help design system level interventions to address the issues facing all kinds of MNS disorders and their respective medicines. Another limitation of the study is that due to inclusion of psychotropic drugs in widest sense, the respondents were not specifically asked to differentiate between psychotropic drugs that pose risk for abuse and that do not. So, this study only provides information for the extend of abuse of psychotropic drugs as a bigger category and it does not provide specific details of drugs with and without abuse potentiality. One of the limitations is that the inter-rater reliability was not explicitly considered as all the researchers worked as a team, from the development of topics guides to the finalization of the coding framework.

## Conclusions

Except strict pre-approval processes, quantity restriction and provision of record keeping, all other aspects of supply chain management of psychotropic drugs are similar to that of general drugs in Nepal. Generally, the quality and effectiveness of psychotropic drugs were perceived to be good, although some respondents thought that the drugs from Indian and multinational companies were of better quality compared to the drugs produced in Nepal. Despite having side effects, many respondents thought psychotropic drugs were effective for mental health problems. However, some respondents also stressed that psychosocial counseling and other forms of social support are equally important to increase the effectiveness of psychotropic drugs. It was widely accepted by the respondents that bonuses and commissions were prominent in Nepal’s drug supply chain which encouraged inappropriate use or misuse of psychotropic drugs. Although psychotropic drugs should be sold only with a prescription, this was often not implemented in practice. The respondents stated that the misuse of psychotropic drugs takes place by prescribers who over-prescribe, by patients who over-dose, and by retailers who profit from illegal sales. The perceived stigma of mental illness forces patients and their family members to discontinue medicines with a fear that others would know. Therefore, both the social stigma towards people with mental health problems and the perceived stigma among those with mental health problems and their families need to be addressed to ensure effective use of psychotropic drugs.

## Additional file

Additional file 1: Table S1. Names of psychotropic drugs available in the Nepalese market. (DOCX 13 kb)

## Abbreviations

DDA: Department of Drug Administration
DoHS: Department of Health Services
DPHO: District Public Health Office
EMERALD: Emerging Mental Health Systems in Low- and Middle-Income Countries
HA: Health Assistant
LMD: Logistics Management Division
LMICs: Low and Middle Income Countries
MBBS: Medicine Bachelor and Bachelor of Surgery
MHBF: Mental Health Beyond Frontiers
mhGAP: Mental Health Gap Action Program
MNS: Mental, Neurological and Substance use disorder
MoH: Ministry of Health
MR: Medical Representative
NGOs: Non-Governmental Organizations
NHRC: Nepal Health Research Council
PHCRD: Primary Health Care Revitalization Department
PRIME: Program for Improving Mental Health Care
RMS: Regional Medical Stores
