# Supplementary material for: Psychotropic drugs in Nepal: perceptions on use and supply chain management
Source: Global Health. 2018 Jan 24;14:10. doi: 10.1186/s12992-018-0322-4 (PMC5784600; doi:10.1186/s12992-018-0322-4)
Supplement: Additional file 1: Table S1. — Names of psychotropic drugs available in the Nepalese market. (DOCX 13 kb) [file 12992_2018_322_MOESM1_ESM.docx]

Supplementary Table: Names of psychotropic drugs available in the Nepalese market

| Psychotropic drugs available in Nepal | Mentioned by the respondents | Included in National Essential Drug List | Included in Free Drug List of MoH | Included in DDA list for compulsory record keeping provisions |
| --- | --- | --- | --- | --- |
| Alprozolam | Yes | Yes | Yes | Yes |
| Amitryptyline | Yes | Yes | Yes | No |
| Chlorpromazine | Yes | Yes | Yes | No |
| Diazepam | Yes | Yes | Yes | Yes |
| Phenobarbitol | Yes | No | Yes | Yes |
|  |  |  |  |  |
| Cholrdiazepoxide | Yes | Yes | No | Yes |
| Buprenorphine | No | Yes | No | Yes |
| Clomipramine | No | Yes | No | No |
| Disulfiram | No | Yes | No | No |
| Fluoxetine | Yes | Yes | No | No |
| Fluphenazine | No | Yes | No | No |
| Haloperidole | Yes | Yes | No | No |
| Lithium Carbonate | Yes | Yes | No | No |
| Methadone | No | Yes | No | Yes |
| Risperidone | Yes | Yes | No | No |
| Thioridazine | No | Yes | No | No |
|  |  |  |  |  |
| Gabapentin | Yes | No | No | No |
| Flurazepam | No | No | No | Yes |
| Glutethiamide | No | No | No | Yes |
| Larazepam | No | No | No | Yes |
| Medazepam | No | No | No | Yes |
| Meprobamate | No | No | No | Yes |
| Midazolam | No | No | No | Yes |
| Midazolam | Yes | No | No | No |
| Nitrozepam | Yes | No | No | Yes |
| Benzhexol | Yes | No | No | No |
| Carbamazepine | Yes | No | No | No |
| Colbazam | No | No | No | Yes |
| Clonazepam | Yes | No | No | Yes |
| Clozapine | Yes | No | No | No |
| Olanzapine | Yes | No | No | No |
| Oxazepam | No | No | No | Yes |
| Oxazolam | No | No | No | Yes |
| Pentazocine | No | No | No | Yes |
| Phentermine | No | No | No | Yes |
| Prazepam | No | No | No | Yes |
| Propranolol | Yes | No | No | No |
| Prometazine | Yes | No | No | No |
| Quitiapine | Yes | No | No | No |
| Sertraline | Yes | No | No | No |
| Trifluperazine | Yes | No | No | No |
| Trihexyphenydyl | Yes | No | No | No |
| Valproic acid | Yes | No | No | No |
| Zolpidem | Yes | No | No | Yes |
| Triazolam | No | No | No | Yes |
